# Supplementary material for: Cryptic MYC insertions in Burkitt lymphoma: New data and a review of the literature
Source: PLoS One. 2022 Feb 15;17(2):e0263980. doi: 10.1371/journal.pone.0263980 (PMC8846522; doi:10.1371/journal.pone.0263980)
Supplement: S1 Fig — (PPTX) [file pone.0263980.s001.pptx]

## Slide 1
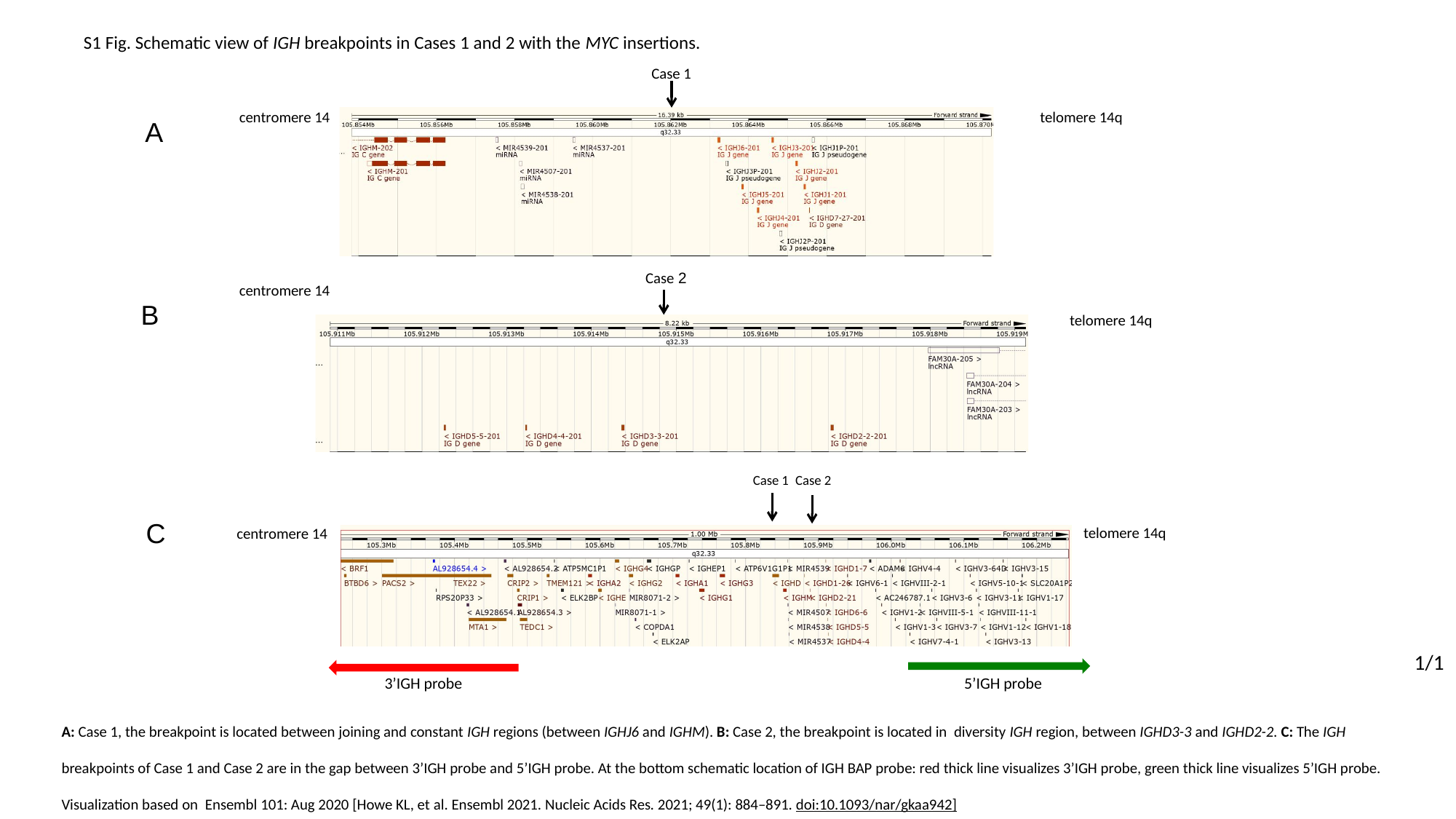

S1 Fig. Schematic view of IGH breakpoints in Cases 1 and 2 with the MYC insertions.
Case 1
telomere 14q
centromere 14
A
Case 2
centromere 14
B
telomere 14q
Case 1
Case 2
C
telomere 14q
centromere 14
3’IGH probe
5’IGH probe
1/1
A: Case 1, the breakpoint is located between joining and constant IGH regions (between IGHJ6 and IGHM). B: Case 2, the breakpoint is located in diversity IGH region, between IGHD3-3 and IGHD2-2. C: The IGH breakpoints of Case 1 and Case 2 are in the gap between 3’IGH probe and 5’IGH probe. At the bottom schematic location of IGH BAP probe: red thick line visualizes 3’IGH probe, green thick line visualizes 5’IGH probe. Visualization based on Ensembl 101: Aug 2020 [Howe KL, et al. Ensembl 2021. Nucleic Acids Res. 2021; 49(1): 884–891. doi:10.1093/nar/gkaa942]
