# Supplementary material for: Cryptic MYC insertions in Burkitt lymphoma: New data and a review of the literature
Source: PLoS One. 2022 Feb 15;17(2):e0263980. doi: 10.1371/journal.pone.0263980 (PMC8846522; doi:10.1371/journal.pone.0263980)
Supplement: S1 Table — (DOCX) [file pone.0263980.s002.docx]

**S1 Table. Summarized data of classical cytogenetic analyses in patients with suspected Burkitt lymphoma.**

| **Karyotype** | **BL**  **(no of cases)** | **BLL,11q**  **(no of cases)** | **BL,*MYC*R*/*11q**  **(no of cases)** |
| --- | --- | --- | --- |
| Total karyotype analysis | 73 | 10 | 5 |
| t(8;14)(q24;q32) | 60 | 0 | 3 |
| t(8;22)(q24;q11) | 7 | 0 | 2 |
| t(2;8)(p11;q24) | 1 | 0 | 0 |
| 11q duplication/deletion | 0 | 10 | 5 |
| Normal karyotype/karyotype without t(8;V) | 5 | 0 | 0 |

BL, Burkitt lymphoma; BLL,11q, Burkitt-like lymphoma with 11q aberration; BL,*MYC*R/11q, Burkitt lymphoma with both the *MYC* rearrangement and 11q gain/loss; 11q duplication/deletion in karyotype is most often described in the literature as 11q gain/loss; t(8;V), translocation of 8q24 (*MYC* locus) and one of the loci: 14q32 (*IGH*), 22q11 (*IGL*), and 2p11 (*IGK*).
