# Supplementary material for: Cryptic MYC insertions in Burkitt lymphoma: New data and a review of the literature
Source: PLoS One. 2022 Feb 15;17(2):e0263980. doi: 10.1371/journal.pone.0263980 (PMC8846522; doi:10.1371/journal.pone.0263980)
Supplement: S1 File — (DOCX) [file pone.0263980.s003.docx]

**Supporting methods**

**Immunohistochemistry**

For many years (in years 2003- 2015), immunohistochemistry (IHC) was performed on deparaffinized tissue sections using Avidin–Biotin Complex (ABC) (ThermoScientific) and the Labeled Streptavidin–Biotin (LSAB) (ThermoScientific) staining methods. Recently (in years 2015-2020) the EnVision Detection Systems FLEX kit (Dako, Carpinteria, CA, USA, code K 8000) or ultra-View Universal DAB Detection kit (Ventana Medical Systems, Tucson, USA, catalog no. 760-500) and, if necessary, antigen-retrieval technique was applied for each monoclonal antibody according to the manufacturer’s instructions. Primary antibodies against the following proteins were used: CD(3/5/10/20/38/ 43/44/56), BCL2, BCL6, LMO2, MYC, MUM1, Tdt, EBV-LMP1 and Ki-67. The source, manufacturer, and working dilutions of the primary antibodies are summarized in  Table 1. Tissue sections were incubated for 1 hour with the diluted antibodies in an autostainer (Dako) (most antibodies) and detected by EnVision FLEX system, but the diluted LMO2, MYC and CD56 (clone: MRQ-42) were incubated for 40 minutes after the antigen retrieval with CC1 solution for 60 minutes, and detected by ultraView Universal DAB Detection Kit in an automated immunostainer (Benchmark XT; Ventana).

Table 1**.** The clones of antibodies used for immunohistochemistry analyses.

| **MoAb** | **CD3** | **CD5** | **CD10** | **CD20** | **CD43** | **CD44** | **BCL 2** | **BCL 6** | **MUM1** | **Ki-67** | **CycD1** | **EBV-LMP1** | **CD56** | **CD56** | **LMO2** | **CD38** | **TdT** | **MYC** |
| --- | --- | --- | --- | --- | --- | --- | --- | --- | --- | --- | --- | --- | --- | --- | --- | --- | --- | --- |
| Clone | F7.2.38 | SP19 | 56C6 | L26 | DF-T1 | DF  1485 | 124 | PG-B6p | MUM1p | MIB-1 | SP4/  EP-12 | CS.1-4 | 123  C3 | MRQ-42 | SP51 | SP149 | B6 | Y69 |
| Dilution | 1:50 | F | F | F | F | 1: 50 | F | F | F | F | F | F | F | 1:100 | 1:100 | 1:100 | 1:20 | 1:100 |
| Source | Dako | | | | | | | | | | | | | CM | | | Novocastra | A |

A, Abcam (Cambridge, United Kingdom); CM, Cell Marque (Rocklin, California, USA); F, FLEX EnVision FLEX; MoAb, monoclonal antibody; Dako, (Glostrum, Denmark); Novocastra Leica Microsystems (Berlin, Germany), High pH is a high-sensitivity visualization system, intended for use in immunohistochemistry together with Dako Omnis autostainer.

**Flow cytometry immunophenotyping analysis**

Immunophenotype was also determined by flow cytometry (FCM) of cellular suspension obtained by fine needle aspiration biopsy (FNAB). Within the context of a single FNAB, four to ten separate needle passes within a lymph node or tumor and three or four passes within abdominal mass provided adequate cellular material. FNAB samples for FCM, were collected into K2-EDTA tubes containing PBS. The trypan blue exclusion test was used to determine the number of viable cells present in a cell suspension during FNAB. Immediately after delivery to the laboratory, 50 µl (<1x10^6^ nucleated cells) of cellular suspension was mixed and incubated (15 min. at room temperature in the dark) with 20 µl of the relevant mouse (or rat) anti-human monoclonal antibodies (MoAbs). FNAB samples were prepared by the standard red blood cell lyse/wash technique with FACSlysing Solution (BD, San Jose, USA) and were stained for 3 or 5-color immunophenotyping. Samples were washed once in PBS solution without Ca/Mg ions and then suspended in 0,5ml of 1% paraformaldehyde in PBS. Burkitt lymphoma (BL) cells were stained with MoAbs with one of the following fluorochromes: FITC/ PE, RPE/PerCP, PerCP-Cy-5.5, PE-Cy7/APC^*^. To establish negative fluorescence signal threshold for each fluorochrome, and to evaluate non-specific binding, all samples were stained with isotype controls corresponding to the MoAb type used in the experiment. An optimum panel of MoAbs and their clones used for the FCM analysis to evaluate BL are listed in Table 2. The intracytoplasmic staining of BCL2 and intranuclear staining of BCL6/MYC was performed following cell permeabilization with PermeaFix (BD). Photomultiplier tube and compensation settings on the FACSCalibur or FACSCanto II cytometers were adjusted daily, with an initial setup with the use of Calibrate beads and FACSComp software (BD). Five thousand events were acquired in a "live gate", covering the CD45(+)/CD14(–) large lymphoid region, including neoplastic cells and normal lymphocytes, as previously published [1,2]. The data was gathered from the flow cytometers and processed using the BD CELLQuest software (BD).

*FITC, PE, RPE, PerCP, PerCP-Cy5.5, PE-Cy7, APC represent fluorescein isothiocyanate, phycoerythrin, R-phycoerythrin, peridinin-chlorophyll protein, PerCP with a cyanine dye (Cy5.5), PE with a cyanine dye (Cy7) and allophycocyanin, respectively.

Table 2**.** Antibodies used for flow-cytometry.

| **MoAb** | **CD45** | **HLA-DR** | **CD19** | |  | | **CD20** | | **CD22** | | **CD23^** | **CD79β** | **FMC7** | **CD3** | **CD4** | **CD5** | **CD8** | **CD25** | **CD54** | **CD56** | **CD16&CD56** | **CD10** | **CD11c** | **CD38*** | **CD62L** | **CD71** | **CD81** | **CD200** | **CD305** | **CD49d** | **κ/λ** | **BCL2** | **BCL6** | **IgG1/IgG2a** | **IgG1** | **CD43** | **CD138** | **CD52** | **CD44** | **IgD, IgG, IgM** | **IgG1** |
| --- | --- | --- | --- | --- | --- | --- | --- | --- | --- | --- | --- | --- | --- | --- | --- | --- | --- | --- | --- | --- | --- | --- | --- | --- | --- | --- | --- | --- | --- | --- | --- | --- | --- | --- | --- | --- | --- | --- | --- | --- | --- |
| Clone | 2D1 | L243 | 4G7 | |  | | L27 | | S-HCL-1 | | EBVCS-5/ M-L233^ | SN8 | FMC7 P3-NS1-1-AG4-1 | SK7 | SK3 | L17F12 | SK1 | 2A3 | LB-2 | NCAM16.2 | B73,1/MY31 | HI10a | S-HCL-3 | HB7, FITC/PE | SK11 | L01.1 | JS-81 | MRC OX-104 | DX26 | 9F10 | TB28-2/1-155-2 | Bcl-2/100 | K112-91 | X40/X39 | MOPC-21 | DFT-1 | B-A38 | YTH34.5 | MEM-85 | N/A | MOPC-21 |
| Source | |  | |  | |  | |  | | BD | | | | | | | | | | | | | | | | | | | | | | | | | | Serotec | | | Invitrogen | | Caltag |

^two different clones of CD23 antibody; *CD38, HB7 clone conjugated with FITC (fluorescein isothiocyanate) and PE (phycoerythrin); BCL2, an intracytoplasmic antigen and BCL6, an intranuclear antigen, were assessed following permeabilization of cells; IgD, IgM, IgG – antibodies against heavy chains; IgG1/IgG2a and IgG1 – antibodies of isotype control conjugated with FITC/PE and APC (allophycocyanin), respectively; κ/λ (kappa/lambda) – antibodies against light chains; MoAb, monoclonal antibodies

**DNA quality assessment**

Before the creation of the NGS libraries, the quality of DNA was evaluated by Real-Time quantitative PCR using the personally developed method based on the comparison of PCR efficiency for two amplicons of different lengths. Two primer pairs specific to the *GAPDH* gene were used. The short PCR product (S, length = 68 bp) was obtained with the following primers: 5’-ATGCTGCATTCGCCCTCTTA-3’ (GAPDH-SF), 5’-GGGGAGGCTCCTCCAGAATA-3’, (GAPDH_SR). The 5’-CAGCCCCTTCATACCCTCAC-3’ (GAPDH_MF) and 5’-CTAGTTGCCTCCCCAAAGCA-3’ (GAPDH_MR) primers were used to get the medium-length PCR product (M, length = 187 bp). Both qPCR reactions were run on the 7500 Fast Real-Time PCR System (Thermo Fisher Scientific, Waltham, MA, USA), in triplicates, in the volume of 10 µl, according to the following thermal profile: 50 ºC for 2 min., 95 ºC for 2 min., 40 cycles including two steps: 95 ºC for 10 s, and 60 ºC for 30 s, followed by the melt curve stage in conditions recommended by Thermo Fisher Scientific. The usage of PowerUp SYBR Green Master Mix with uracil N-glycosylase (UNG) and dUTP (Thermo Fisher Scientific) allowed for monitoring of the amounts of obtained PCR products in real-time and also prevented contamination with carry-over PCR products. The amplification efficiencies of the short and the medium-length amplicon were calculated based on the relevant standard curves generated from a good-quality control DNA of known concentration assessed with the QuantStudio 3D Digital PCR System (Thermo Fisher Scientific). The amounts of both PCR products were also compared to each other to get the M/S ratio. This ratio ranged from 0 to 1 and its value was positively correlated with the DNA quality. Samples with the M/S ratio > 0.05 were found suitable for the NGS analysis.

***In vitro* verification of the CTX found *in silico***

For each case, the PCR reactions were performed with the AmpliTaq Gold DNA polymerase (Thermo Fisher Scientific), and they shared the same thermal profile: 95 ºC for 5 min, followed by 40 cycles consisting of denaturation (94 ºC for 30 s.), primers annealing (65 ºC for 30 s.), and product elongation (72 ºC for 45 s.). Final elongation step in 72 ºC lasted for 5 min. Then, the samples were cooled down to 4 ºC. The PCR products were first analyzed by agarose gel electrophoresis with the Midori Green Advance DNA Stain (Nippon Genetics Europe GmbH, Düren, Germany). Then, the PCR products were Sanger sequenced on the 3500 Genetic Analyzer using the BigDye Terminator v3.1 Cycle Sequencing Kit (both manufactured by Thermo Fisher Scientific) and the corresponding reverse primer. The thermal profile of both sequencing reactions was as follows: 95 ºC for 2 min., 35 cycles involving: 96 ºC for 10 s, 65 ºC for 5 s., 70 ºC for 4 min. Finally, the samples were cooled down to 4 ºC.

**Supporting references**

1. Rymkiewicz G, Grygalewicz B, Chechlinska M, Blachnio K, Bystydzienski Z, Romejko-Jarosinska J, et al. A comprehensive flow-cytometry-based immunophenotypic characterization of Burkitt–like lymphoma with 11q aberration. Mod Pathol. 2018; 31: 732–743. doi:10.1038/modpathol.2017.186.

2. Zajdel M, Rymkiewicz G, Chechlinska M, Blachnio K, Pienkowska-Grela B, Grygalewicz B, et al. miR expression in MYC-negative DLBCL/BL with partial trisomy 11 is similar to classical Burkitt lymphoma and different from diffuse large B-cell lymphoma. Tumour Biol. 2015; 36: 5377–5388. doi:10.1007/s13277-015-3203-y.
