# Supplementary material for: Cryptic MYC insertions in Burkitt lymphoma: New data and a review of the literature
Source: PLoS One. 2022 Feb 15;17(2):e0263980. doi: 10.1371/journal.pone.0263980 (PMC8846522; doi:10.1371/journal.pone.0263980)
Supplement: Attachment — Submitted filename: Response_to_Reviewers.docx [file pone.0263980.s005.docx]

Dear Prof. Vincenzo L’Imperio,

We appreciate you and the reviewers for your precious time in reviewing our paper and

providing valuable comments once again. All comments were helpful for revising and improving our paper and we have taken them fully into account in revision.

We are submitting the corrected manuscript with the suggestions incorporated to the text.

Our responses to all the comments are as follows:

(All page and line numbers refer to the manuscript file without track changes - file name “Manuscript”)

**Comments from Reviewer #2:**

**Comment 1: Page 4, lines 84-87: this part about epidemiological data of the population should be reported in the results section.**

Response: Thank you for suggestion. According to the Reviewer's request, we have transferred the part about epidemiological data from Materials and methods to the Results section (pages 8, lines 169-172 in the revised version of manuscript):

“This group of patients consisted of 102 adults with median age of 35 years (range, 19-79 years) and 6 children with median age of 8 years (range, 3-12 years). Among adult patients, 81 were male and 21 were female (ratio, 3.86:1). Among pediatric patients, 5 were male and 1 was female (ratio, 5:1).”

**Comment 2: Figure 6: the diagrams are of low quality, small and difficult to read.**

Response: According to the Reviewer's #2 suggestion, the diagrams (immunophenotype descriptions) in Figure 6, which were of low quality, small and difficult to read, have been changed in the revised manuscript as requested. We decided to change the colour (from blue to black), and to increase the font size to get a higher quality figure. In a similar way, we changed the font of Figure 3.

**Comment 3: Page 15, line 326; page 16, line 354 and figure 6: you should check the agreement between BCL6 flow cytometry results in the text and in figure 6 description and diagrams.**

**Comment 4: Page 15, line 326: “BL cells from the peritoneal fluid and liver tumor were positive for…BCL6”**
**Comment 5: Page 16, line 354 and figure 6: “E-I: BL expresses a homogeneous phenotype of germinal center origin (CD81+higher/CD10+/CD38+higher/CD44–) but BCL6 negative”.**

Response to comments 3, 4, 5: We sincerely apologize for the error in the text describing the BL immunophenotype (**Page 15, line 326: “BL cells from the peritoneal fluid and liver tumor were positive for…BCL6”)** in terms of BCL6 expression in the flow cytometric test. A fragment of the text concerning BCL6 flow cytometry results has been changed according to Reviewer#2 suggestion (pages 14-15, lines 312-318 in the revised manuscript):

“BL cells from the peritoneal fluid and liver tumor were positive for CD45^weaker^/CD20^bright^/CD19^bright^/CD22 (with an order according to MFI of CD20 > CD19 > CD22)/ CD10/CD38^higher^ (with an MFI of 873 for CD38, compared to an MFI of 36 on T lymphocytes) /CD81^higher^/CD79β/HLA-DR/CD43^weaker^/CD49d^weaker^/CD52^higher^/CD54^higher^/CD305/MYC and surface immunoglobulin (IgD/IgM), while they were negative for CD5/CD8/CD11c/CD23/CD25/CD44/ CD16&CD56/CD56/CD62L/CD200/IgG/λ/κ and BCL6.”

We would like to thank the referee again for taking the time to review our manuscript.

Sincerely,

Renata Woroniecka

25-01-2022
